# Fungal Strains as Catalysts for the Biotransformation of Halolactones by Hydrolytic Dehalogenation with the Dimethylcyclohexane System

**DOI:** 10.3390/molecules17089741

**Published:** 2012-08-14

**Authors:** Małgorzata Grabarczyk

**Affiliations:** Department of Chemistry, University of Environmental and Life Sciences, Norwida 25, 50-375 Wrocław, Poland; Email: magrab@onet.pl; Tel.: +48-71-3205-252; Fax: +48-71-3207-744

**Keywords:** lactones, biotransformation, hydrolytic dehalogenation, *Fusarium* species

## Abstract

Bicyclic chloro-, bromo- and iodo-γ-lactones with dimethylcyclohexane rings were used as substrates for bioconversion by several fungal strains (*Fusarium*, *Botrytis* and *Beauveria*). Most of the selected microorganisms transformed these lactones by hydrolytic dehalogenation into the new compound *cis*-2-hydroxy-4,6-dimethyl-9-oxabicyclo[4.3.0]- nonan-8-one, mainly the (−)-isomer. When iodo-γ-lactone was used as the substrate, two products were observed: a hydroxy-γ-lactone and an unsaturated lactone. The structures of all substrates and products were established on the basis of their spectral data. The mechanism of dehalogenation of three halolactones was also studied.

## 1. Introduction

Halogenated organic compounds are often found in Nature. They are typically isolated from many marine organisms such as red algae, bacteria, sponges and corals. Among them there are found sesquiterpenoids [1,2,3], diterpenoids [4,5,6], lactones [7,8] and macrolides [9,10]. Halocompounds can be used as therapeutic agents and they have different biological properties such as anticancer [11,12], cytotoxic [13,14,15,16,17], antimicrobial [18] and antimalarial [19] activities. Halogenated organic compounds are also used as solvents, as intermediates for chemical syntheses and also as pesticides or artificial fertilisers. Such compounds are theoretically good because they protect our food from pests and they have a positive effect on crop growth, but they can accumulate in soil or in water. As a consequence, in large amounts, they can be poisonous to other animals or fish [20,21].

There are many different methods used for the biodegradation of halocompounds containing aromatic rings using algae [22,23], bacteria [24,25,26], fungi [27,28,29,30]. There are fewer reports about dehalogenation of non-aromatic compounds. Bacteria have been used to biodegrade different aliphatic compounds [31,32,33]. Various fungal strains were used for biodegradation of terpenoid halo-lactones [34,35,36]. These fungal strains are mainly able to introduce a hydroxy group in the place of the halogen atom or to eliminate the halogen through the formation of a double bond.

Filamentous fungi of the genus *Fusarium*, *Beauveria* and *Botrytis* are known for their hydrolytic properties in relation to miscellaneous compounds. Filamentous fungi of the genus *Fusarium* were often used for biohydroxylation of different substrates such as sesquiterpenoids [37], diterpenoids [37,38,39,40,41], steroids [42], sesquiterpene lactones [43,44,45], nitriles [46] and flavonoids [47]. An interesting example of biohydroxylation is the use of *Fusarium oxysporum* for the production of α-terpineol (a commonly used fragrance compound) [48,49]. The fungal strain *Beauveria bassiana* was used for biohydroxylation of hydrocarbons [50], sesquiterpenes [50,51], diterpenes [52], lactone lovastatin [53] amides [50]. The filamentous fungus *Botrytis cinerea* was also used to introduce hydroxy groups on different substrates like monoterpenes [37], sesquiterpenoids [54,55], steroids [54] or halogenated aromatic compound [56].

Bearing in mind that the fact that there are many reports about biological hydroxylation, but very few of them concern the hydrolytic dehalogenation of halolactones, therefore, in this study, further examples of the biohydroxylation of chloro-, bromo- and iodolactones with a dimethylcyclohexane ring are shown. Halolactones have been chosen as model compounds because some of them exhibit antifeeding activity against different insect pests and they can be used in insect pest control [57,58,59,60,61,62]. Additionally, halolactones are relatively simple to synthesise in good yield. Using these lactones as substrates led me to select microorganisms which are able to eliminate a halogen atom from a molecule. An additional advantage of this method is that fungal strains often introduce a hydroxy group into the molecule, providing hydroxylactones which are different than those obtained using the classical synthetic methods.

## 2. Results and Discussion

**Scheme 1:**
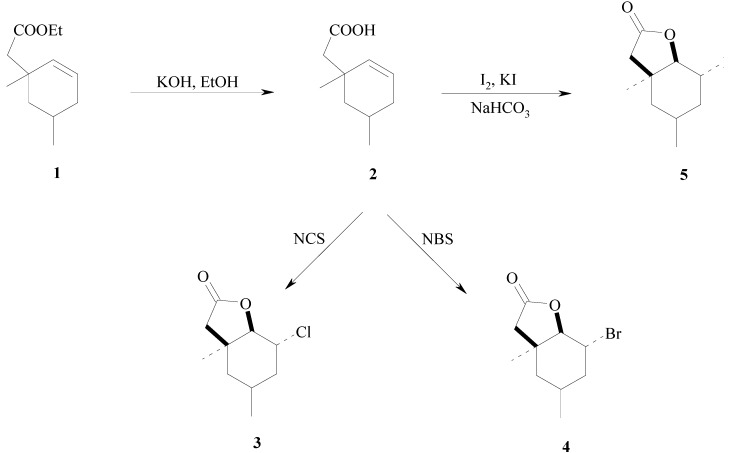
Synthesis of halolactones **3**–**5**.

The substrates used for biotransformation were the racemic halolactones **3**–**5**. They were all synthesised from a known γ,δ-unsaturated ester **1** (Scheme 1). Iodolactone **5** was obtained by basic hydrolysis of ester **1** and iodolactonisation of acid **2** according to the procedure described earlier [63]. Lactones **3** and **4** were also obtained in two steps: basic hydrolysis of ester **1** and bromo- or chlorolactonization using *N*-bromosuccinimide (NBS) or *N*-chlorosuccinimide (NCS), respectively.

The structures of chloro-γ-lactone **3** and bromo-γ-lactone **4** were determined on the basis of their spectral data (^1^H-NMR, ^13^C-NMR, COSY, HMQC and IR) and elemental analysis. The absorption bands at 1788 cm^−1^ for **3** and 1789 cm^−1^ for **4** in their IR spectra confirmed the presence of a γ-lactone ring in the structure of these compounds. The similarity between the spectral data (^1^H-NMR) of chlorolactone **3**, bromolactone **4** and the previously obtained iodolactone **5** [63] suggested that their structures were identical. In all three cases, the cyclohexane ring was found in the chair conformation. The signal of proton H-1 in the NMR spectra looked like a broad singlet. The multiplet of proton H-2 had very small coupling constants. This indicates that both H-1 and H-2 protons were in *trans*-diequatorial positions.

Biotransformations were carried out in two steps: first, the screening transformation and then the preparative one. During the screening procedure, eight fungal strains of local origin: *Fusarium culmorum*, *Fusarium avenaceum*, *Fusarium oxysporum*, *Fusarium tricinctum*, *Fusarium semitectum*, *Fusarium solani*, *Botrytis cinerea* and *Beauveria bassiana* were tested for their ability to convert halolactones into other products. The progress of all the biotransformations was monitored by means of standard techniques (TLC and GC). All known products (**1**, **2**, **5** and **7**) obtained during syntheses or biotransformation were analyzed on a HP-5 column on the basis of their retention times due to with their known patterns obtained earlier in our team [63]. The retention times of new and expected products (**3** and **4**) were compared with the analogous ones obtained previously [35]. During the biotransformation of chloro- **3** and bromolactone **4** only two peaks coming from the substrate and the product were observed in the chromatogram, whose relationship changed over time. The structures of all obtained products were then confirmed by spectroscopic methods (^1^H-NMR, ^13^C-NMR, COSY, HMQC and IR) and also elemental analysis.

**Scheme 2:**
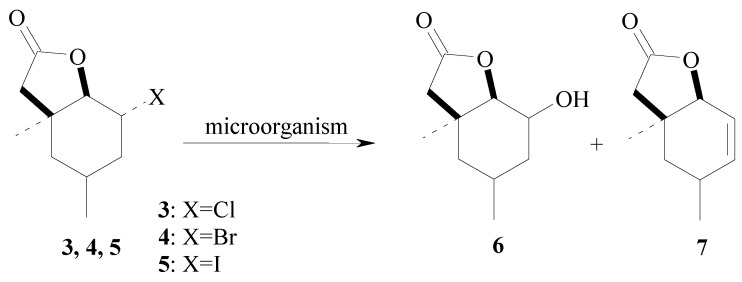
Biotransformations of halolactones **3**–**5**.

The composition of the product mixture shows that, when chloro- **3** and bromolactone **4** were used as substrates, hydroxylactone **6** was obtained as the only product. During the transformation of iodolactone **5**, the formation of the same hydroxylactone **6** was observed and in four cases (when *F. culmorum*, *F. solani*, *F. avenaceum* and *F. semitectum* were used as biocatalysts) the unsaturated lactone **7** [63] (Scheme 2, Table 1) was obtained too.

**Table 1:** The composition (in % according to GC) of the product mixtures of screening biotransformations of lactones **3**, **4**, **5**.

Strain	Time of incubation (days)	Products of transformations (%)					
		6 (from 3)		6 (from 4)		6 (from 5)	7 (from 5)
*Fusarium culmorum* AM10	5	-		19.5		10.3	28.5
	9	5.0		54.1		22.7	41.2
	14	7.4		**75.1**		**35.5**	**51.8**
*Fusarium avenaceum* AM11	5	-		7.8		4.8	1.4
	9	27.4		48.2		10.4	2.9
	14	**57.7**		**56.3**		15.9	6.3
*Fusarium oxysporum* AM13	5	9.1		1.5		2.5	-
	9	40.5		27.6		36.3	-
	14	**60.4**		**65.5**		**52.0**	-
*Fusarium tricinctum* AM16	5	-		10.8		3.1	-
	9	20.9		24.7		16.4	-
	14	**53.3**		52.4		24.8	-
*Fusarium semitectum* AM20	5	5.7		6.6		10.5	11.0
	9	16.7		30.3		22.7	20.0
	14	51.4		**55.9**		42.0	9.3
*Fusarium solani* AM203	5	1.8		27.7		2.7	-
	9	33.0		48.7		35.8	6.5
	14	**76.6**		**61.1**		**54.7**	7.7
*Botrytis cinerea* AM235	5	3.3		3.6		7.3	-
	9	84.1		56.6		24.3	-
	14	**85.3**		**95.0**		27.7	-
*Beauveria bassiana* AM278	5	6.1		3.3		3.5	-
	9	22.3		18.7		10.2	-
	14	29.7		26.5		19.7	-

These results indicated that all three substrates, chloro- **3**, bromo- **4** and iodolactone **5**, were always transformed into the same product—hydroxylactone **6**. An additional product (unsaturated lactone **7**) was observed only for iodolactone **5**, in three cases in small quantities and in one (when *Fusarium culmorum* was used) in good yield. Two microorganisms, *Fusarium oxysporum* and *Fusarium solani*, transformed all three substrates with good yields (respectively 52%–60% and 55%–77%). Using *Botrytis cinerea* led to products from chloro- and bromolactone with high yield (85% and 95%). The other fungal strains converted one or two substrates with satisfactory yield (between 52% and 75%). During these screening transformations, only the percentage of conversion was checked.

For all three lactones (compounds **3**, **4** and **5**), the abiotic control was carried out by adding each one of them to a sterile medium containing 3 g glucose and 1 g peptobac in water (100 mL) and shaking for two weeks. The samples were taken as in the case of the screening procedure. As a result of the experiments, it was found that only the substrate was always in the reaction mixture. This means that the hydrolytic dehalogenation of these halolactones does not occur without the presence of the microorganisms. Another experiment was then carried out. The fungal strains were incubated for 14 days without the addition of substrate. This allowed me to find the secondary metabolites produced by the tested strains.

The next step of preparative biotransformations was to find fungal strains which were capable of converting the substrates in yields over 50%. Different *Fusarium* species and *Botrytis cinerea* were chosen for this purpose. The results of these transformations are shown in Table 2, Table 3, Table 4.

The structures of the two products **6** and **7** were established on basis of their spectral data. The IR spectra showed that the γ-lactone ring had been retained during the biotransformation in both products (absorption bands at 1788 cm^−1^ and 1779 cm^−1^, respectively). A strong, broad band found at 3292 cm^−1^ for **6** suggested the presence of a hydroxy group in the molecule.

**Table 2:** Results of preparative biotransformations of lactone **3** after 14 days.

Strain	3 (%)	6 (%)	Isolated yield (%)	Isolated yield (g)	*ee* (%)	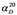
*F. avenaceum * AM11	43.2	56.8	31.8	0.029	6.7	+6.92 ( *c *= 1.06, CHCl_3_)
*F. oxysporum * AM13	39.0	61.0	26.3	0.024	15.8	−7.45 ( *c *= 0.93, CHCl_3_)
*F. tricinctum * AM16	25.8	74.2	36.2	0.033	25.8	−14.12 ( *c *= 0.60, CHCl_3_)
*F. solani * AM203	12.2	87.8	37.3	0.034	33.8	−17.46 ( *c *= 0.59, CHCl_3_)
*B. cinerea * AM235	16.2	83.8	37.3	0.034	16.7	−9.40 ( *c *= 0.32, CHCl_3_)

**Table 3:** Results of preparative biotransformations of lactone **4** after 14 days.

Strain	4 (%)	6 (%)	Isolated yield (%)	Isolated yield (g)	*ee* (%)	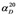
*F. culmorum* AM10	22.7	77.3	31.7	0.024	0	-
*F. avenaceum* AM11	41.6	58.4	30.2	0.023	12.8	−4.56 ( *c *= 0.59, CHCl_3_)
*F. oxysporum* AM13	33.0	67.0	28.6	0.021	19.0	−9.44 ( *c *= 0.77, CHCl_3_)
*F. semitectum* AM20	37.3	62.7	43.0	0.032	16.2	−7.11 ( *c *= 0.94, CHCl_3_)
*F. solani* AM203	33.7	66.3	38.0	0.028	32.0	−33.42 ( *c *= 1.00, CHCl_3_)
*B. cinerea* AM235	7.3	92.7	59.0	0.044	8.9	−2.20 ( *c *= 0.99, CHCl_3_)

**Table 4:** Results of preparative biotransformations of lactone **5** after 14 days.

Strain	4 (%)	6 (%)	7 (%)	Isolated yield of 6 (%)/7 (%)	Isolated yield of 6 (g)/7 (g)	*ee* of 6 (%)/7 (%)	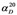
*F. culmorum * AM10	15.2	39.9	44.9	5.8/10.9	0.004/0.006	15.3/0	−15.83 ( *c *= 0.28, CHCl_3_)/-
*F. oxysporum * AM13	40.2	59.8	-	21.2/-	0.016/-	12.3/-	−11.22 ( *c *= 0.21, CHCl_3_)
*F. solani * AM203	37.4	55.5	-	11.1/-	0.008/-	25.1/-	−33.42 ( *c *= 0.38, CHCl_3_)

During the earlier analysis of the structure of iodolactone **5**, it was shown that the signals of the H-1 and H-2 protons were multiplets with very small coupling constants, indicating their *trans* diequatorial positions and the *trans* diaxial positions of the iodine atom and the C-O bond.

The analysis of the ^1^H-NMR spectrum of hydroxy-γ-lactone **7** in comparison to halolactones **3**, **4** and **5** led to the confirmation that the H-1 proton, which was a doublet with a small coupling constant (*J* = 3.1 Hz), was located in an equatorial position. However, the H-2 proton gave a wide mutiplet, which suggested its axial position.

For the determination of the changes in structure of the hydroxylactone **6** relative to the iodolactone **5**, two additional ^1^H-NMR spectra were recorded: COSY and HMQC. The analysis of these spectra led to the determination of differences between these two lactones. In the case of hydroxylactone **6**, some proton signals moved toward a higher field, especially the H-1, H-2 and H-3 protons. The signal of the H-1 proton moved from 4.55 ppm (for iodolactone **5**) to 4.25 ppm (for hydroxylactone **6**), while the signal of the axial H-3 proton moved from 1.59 ppm (for **5**) to 1.22 ppm (for **7**). The biggest difference was visible for the signal from the H-2 proton, which changed its location from 4.70 ppm (for **5**) to 3.80 ppm (for **6**). The shape of the signal of the H-2 proton gave a wide mutiplet which suggested its axial position. The doublet of the H-1 proton with a small coupling constant (*J* = 3.1 Hz) indicated its equatorial position. In the case of the H-3 proton, the signal was a doublet of doublets of doublets for lactone **5** and a doublet of doublets for lactone **6**; the coupling constants changed from 15.3, 11.5 and 3.9 Hz for iodolactone **5** to 12.4 and 12.2 Hz for hydroxylactone **6**. 

**Scheme 3:**
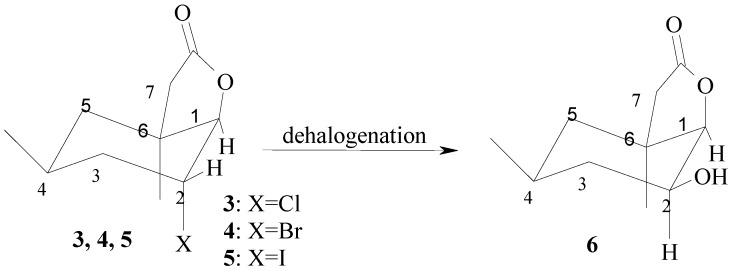
Equatorial location of hydroxy group in lactone **6** as the stereochemical consequence of microbial hydrolytic dehalogenation of chloro- (**3**), bromo- (**4**) and iodolactone (**5**) proceeding with the inversion at C-2.

These observations confirmed that in hydroxylactone **6**, the H-2 proton and the C-O bond were in *trans* diaxial positions, while the H-1 proton and the OH group were situated in *trans* diequatorial positions. These results indicate that the OH group was *cis* oriented in relation to the lactone ring. It suggested also that mechanism of the reaction of the hydrolytic dehalogenation is similar to the S_N_2 substitution (Scheme 3).

The second product of biotransformation of iodolactone **5** was an unsaturated lactone **7**. It was identified due to the presence of the characteristic signal from a double bond (between 5.80 and 5.95 Hz) on the ^1^H-NMR spectra.

The optical activity of all hydroxylactones obtained from halolactones during the preparative biotransformations was measured. The enantioselectivity of biotransformation is taken under consideration to evaluate the ability of microorganisms to transform selected substrates into new products. Enantiomeric excess was determined by GC using a chiral column (Beta Dex, 30 m × 0.25 mm × 0.25 μm). The results are shown in Table 2, Table 3, Table 4. In almost every case, the (−) isomer of lactone **6** was created. Only when chlorolactone **3** was transformed by *F. avenaceum* was the (+) isomer of **6** observed; however, when bromolactone **6** was transformed by *F. culmorum*, the product was a racemic mixture. The enantiomeric excess for all products was very low (from 6.7% to 33.8%). The best result (ee = 25.1% to 33.8%) was observed when *F. solani* was used as the biocatalyst.

## 3. Experimental

### 3.1. General

The progress of chemical reactions and biotransformations and the purity of isolated products were monitored by the TLC technique on silica gel-coated aluminium plates (DC-Alufolien Kieselgel 60 F254, Merck) and by GC analysis which was carried out on a Varian CP-3380 instrument using an HP-5 column (cross-linked methyl silicone gum, 30 m × 0.32 mm × 0.25 µm). The temperatures during GC analysis were as follows: injector 150 °C, detector (FID) 300 °C, column temperature: 100 °C (hold 1 min), 100–200 °C (rate 10 °C/min), 200–300 (rate 50 °C/min), 300 °C (hold 1 min). The enantiomeric compositions of the products obtained during biotransformation were determined by GC analysis using the chiral column CP-cyclodextrin-B-325 (30 m × 0.25 mm × 0.25 µm) under the following conditions: injector 200 °C, detector (FID) 250 °C, column temperature: 140 °C (hold 45 min), 140–200 °C (rate 20 °C /min), 200 °C (hold 1 min). All products were purified by means of preparative column chromatography on silica gel (Kieselgel 60, 230−400 mesh). ^1^H-NMR and ^13^C- NMR spectra were recorded in a CDCl_3_ solution on a Bruker Avance DRX 300 spectrometer. The assignments of ^13^C-NMR chemical shifts were made by means of C/H correlation heteronuclear multiple quantum coherence (HMQC). IR spectra were determined using FTIR on a Thermo-Mattson IR 300 spectrometer. Optical rotations were measured on an Autopol IV automatic polarimeter (Rudolph). Elemental analysis was done using Vario EL III CHNS automatic analyser from Elemental Analyzersysteme.

*2-Chloro-4,6-dimethyl-9-oxabicyclo*[4.3.0]*nonan-8-one* (**3**). A mixture of acid **2** (3.2 g, 0.019 mol) and NCS (3.8 g, 0.029 mol) in THF (30 mL) was stirred at room temperature. After 24 h, water was added to the mixture and the product was diluted with diethyl ether. The separated organic layer was washed with a saturated NaHCO_3_ solution and brine, then dried with anhydrous magnesium sulfate. The crude product was purified on silica gel (hexane-acetone, 3:1) and 2.1 g (yield 50%) of chlorolactone **3** was obtained. ^1^H-NMR (CDCl_3_): 0.93 (d, *J* = 6.5 Hz, 3H, CH_3_C-4), 1.06 (dd, *J* = 13.1 and 13.0 Hz, one of CH_2_-5), 1.39 (s, 3H, CH_3_C-6), 1.53 (m, 1H, one of CH_2_-5), 1.62 (ddd, *J* = 15.2, 12.0 and 3.5 Hz, 1H, one of CH_2_-3), 1.92 (m, 1H, one of CH_2_-3), 2.05 (m, 1H, H-4), 2.26 and 2.48 (two d, *J* = 16.7 Hz, 2H, CH_2_-7), 4.29 (m, 1H, H-1), 4.50 (m, 1H, H-2); ^13^C-NMR (CDCl_3_): 21.25 (C-9), 21.14 (C-4), 23.47 (C-10), 36.48 (C-3), 39.06 (C-6), 41.83 (C-5), 46.43 (C-7), 54.70 (C-2), 84.06 (C-1), 174.69 (C-8); IR (KBr, cm^−1^): 2958 (s), 1788 (s), 1375 (m), 1207 (s), 1009 (m), 670 (m). Anal. Calcd. for C_10_H_15_ClO_2_ (202.68): C, 59.26; H, 7.46. Found: C, 59.18; H, 7.43.

*2-Bromo-4,6-dimethyl-9-oxabicyclo*[4.3.0]*nonan-8-one* (**4**). Acid **2** (3.2 g, 0.019 mol) was dissolved in THF (30 mL), then NBS (4.5 g, 0.025 mol) was added. The mixture was stirred for 24 h at room temperature and then water was added. The product was extracted with diethyl ether. The ether fractions were combined, washed with a saturated NaHCO_3_ solution and brine, then dried with anhydrous magnesium sulfate. The crude product was purified on silica gel (hexane-acetone, 3:1) giving 2.3 g (yield 92%) of bromolactone **4**. ^1^H-NMR (CDCl_3_): 0.95 (d, *J* = 6.5 Hz, 3H, CH_3_C-4), 1.07 (dd, *J* = 16.1 and 13.0 Hz, one of CH_2_-5), 1.45 (s, 3H, CH_3_C-6), 1.56 (dm, *J* = 16.1 Hz, 1H, one of CH_2_-5), 1.70 (ddd, *J* = 15.2, 11.7 and 3.5 Hz, 1H, one of CH_2_-3), 1.97 (m, 1H, one of CH_2_-3), 2.09 (m, 1H, H-4), 2.27 and 2.48 (two d, *J* = 16.7 Hz, 2H, CH_2_-7), 4.43 (m, 1H, H-1), 4.57 (m, 1H, H-2); ^13^C-NMR (CDCl_3_): 21.30 (C-9), 21.88 (C-4), 24.15 (C-10), 37.52 (C-3), 39.15 (C-6), 40.68 (C-2), 41.95 (C-5), 46.62 (C-7), 85.33 (C-1), 174.38 (C-8); IR (KBr, cm^−1^): 2954 (s), 1789 (s), 1371 (m), 1204 (s), 1002 (s), 632 (m). Anal. Calcd. for C_10_H_15_BrO_2_ (247.13): C, 48.60; H, 6.12. Found: C, 48.57; H, 6.17.

*2-Iodo-4,6-dimethyl-9-oxabicyclo*[4.3.0]*nonan-8-one* (**5**). ^1^H-NMR (CDCl_3_): 0.97 (d, *J* = 6.6 Hz, 3H, CH_3_C-4), 1.09 (dd, *J* = 13.2 and 12.9 Hz, one of CH_2_-5), 1.50 (m, 1H, one of CH_2_-5), 1.53 (s, 3H, CH_3_C-6), 1.59 (ddd, *J* = 15.3, 11.5 and 3.9 Hz, 1H, one of CH_2_-3), 1.97 (m, 1H, one of CH_2_-3), 2.07 (m, 1H, H-4), 2.25 and 2.47 (two d, *J* = 16.8 Hz, 2H, CH_2_-7), 4.55 (m, 1H, H-1), 4.70 (m, 1H, H-2); ^13^C-NMR (CDCl_3_): 21.34 (C-9), 33.41 (C-4), 23.77 (C-2), 25.39 (C-10), 38.69 (C-3), 39.62 (C-6), 42.02 (C-5), 46.72 (C-7), 85.79 (C-1), 173.98 (C-8); IR (film, cm^−1^): 2924 (s), 1776 (s), 1453 (m), 1140 (s), 989 (s), 615 (s).

*2-Hydroxy-4,6-dimethyl-9-oxabicyclo*[4.3.0]*nonan-8-one* (**6**). ^1^H-NMR (CDCl_3_): 0.94 (d, *J* = 6.6 Hz, 3H, CH_3_C-4), 1.02 (d, *J* = 13.2 Hz, H-5_ax_), 1.19 (s, 3H, CH_3_C-6), 1.22 (dd, *J* = 12.4 and 12.2 Hz, 1H, H-3_ax_), 1.41 (m, 1H, H-5_eq_), 1.60 (m, 1H, H-4), 1.83 (dm, *J* = 12.4 Hz, 1H, H-3_eq_), 2.00 (m, 1H, OH), 2.31 and 2.46 (two d, *J* = 16.5 Hz, 2H, CH_2_-7), 3.80 (m, 1H, H-2), 4.24 (d, *J* = 3.3 Hz, 1H, H-1); ^13^C-NMR (CDCl_3_): 21.65 (C-10), 21.39 (C-9), 26.88 (C-4), 37.41 (C-3), 40.54 (C-6), 41.21 (C-5), 46.25 (C-7), 68.07 (C-2), 85.81 (C-1), 1753.56 (C-8); IR (film, cm^−1^): 3340 (s), 2912 (s), 1788 (s), 1134 (s), 1052 (s). Anal. Calcd. for C_10_H_15_O_3_ (184.24): C, 65.20; H, 8.75. Found: C, 65.15; H, 8.71.

*4,6-Dimethyl-9-oxabicyclo*[4.3.0]*non-2-en-8-one* (**7**). ^1^H-NMR (CDCl_3_): 1.04 (d, *J* = 7.1 Hz, 3H, CH_3_C-4), 1.15 (s, 3H, CH_3_C-6), 1.22 (dd, *J* = 13.0 and 6.4 Hz, 1H, one of CH_2_-5), 1.52 (dd, *J* = 13.0 and 4.9 Hz, 1H, one of CH_2_-5), 2.24 (m, 1H, H-4), 2.33 and 2.52 (dd, *J* = 17.2 Hz, 2H, CH_2_-7), 4.31 (d, *J* = 4.3 Hz, 1H, H-1), 5.83 (ddd, *J* = 10.0, 4.3 and 2.4 Hz 1H, H-3), 5.94 (d, *J* = 10.0 Hz, 1H, H-2); IR (film, cm^−1^): 1780 (s), 1651 (m), 1212 (s), 999 (s).

### 3.2. Microorganisms

The fungal strains, which were used in this study, were obtained from the collection of the Institute of Biology and Botany, Medical University, Wrocław (*Fusarium culmorum* AM10, *Fusarium avenaceum* AM11, *Fusarium oxysporum* AM13, *Fusarium tricinctum* AM16, *Fusarium semitectum* AM20, *Fusarium solani* AM203, *Botrytis cinerea* AM235 and *Beauveria bassiana* AM278). They all are available in Department of Chemistry, University of Environmental and Life Sciences. These strains were cultivated on Sabouraud’s agar containing 5 g of aminobac, 5 g of peptone, 40 g of glucose and 15 g of agar dissolved in 1 L of distilled water at 28 °C and stored in refrigerator at 4 °C.

### 3.3. Screening Biotransformation

In all transformation experiments, the fungal strains were cultivated on a rotary shaker at 25 °C in two Erlenmeyer flasks which contained 100 mL of medium containing 3 g of glucose and 1 g of peptobac in water (100 mL). After 4 days, 10 mg of the substrate dissolved in 1 mL of acetone was added to the culture in each flask. Incubation of the shaken cultures with the substrate was continued for 14 days. After 5, 9 and 14 days of incubation, 30 mL of the reacting mixture including mycelium, unreacted substrate and expected product was taken from the two flasks and extracted with 30 mL of dichloromethane. After evaporated the solvent the residue was dissolved in 2 mL of acetone and analyzed by TLC (silica gel, hexane-acetone, 3:1) and GC (HP-5 column).

### 3.4. Preparative Biotransformation

The halolactones **3**–**5** (100 mg) were dissolved in 10 mL of acetone and distributed between 10 Erlenmeyer flasks with the 4-day cultures of fungal strains prepared as described in the screening procedure. The selected microorganisms were incubated with the substrates for 14 days, then the products were extracted with dichloromethane (3 × 50 mL). The organic solutions were dried (MgSO_4_) and the solvent was evaporated *in vacuo*. Mixtures containing the product, the unreacted substrate and metabolites of the fungi were separated by column chromatography (silica gel, hexane:acetone 3:1) to obtain pure products.

## 4. Conclusions

The filamentous fungal strains chosen for the conversion of halolactones **3**, **4** and **5** showed very high regio- and stereoselectivity. The OH group was introduced at C-2 in the equatorial position. Since the halogen atom was in an axial position in all three substrates, this allowed us to conclude that the hydrolytic dehalogenation reaction proceeded analogously to the mechanism of S_N_2 nucleophilic substitution. Two substrates, chlorolactone **3** and bromolactone **4**, were transformed by five and six fungal strains, respectively, in good yield (conversions over 50%). The iodolactone **5** was transformed by only three microorganisms in yields over 50%, and additionally this substrate gave an unsaturated lactone **7** in some cases. The best microorganisms for the transformation of halolactones **3**, **4** and **5** in the best yields were *F. solani*, *F. oxysporum* and *B. cinerea*. Taking into account its ability to transform substrates with high enantiomeric excess, the best biocatalyst was *F. solani*. 
